# First detection of *Leishmania infantum* (Kinetoplastida: Trypanosomatidae) in *Culicoides* spp. (Diptera: Ceratopogonidae)

**DOI:** 10.1186/1756-3305-7-51

**Published:** 2014-01-25

**Authors:** Darine Slama, Najoua Haouas, Latifa Remadi, Habib Mezhoud, Hamouda Babba, Emna Chaker

**Affiliations:** 1 Clinical Biology Department, Laboratory of Medical and Molecular Parasitology-Mycology code LR12ES08, Faculty of Pharmacy, Monastir, Tunisia; 2 Laboratory of Parasitology and Mycology, La rabta, Tunis, Tunisia; 3 Laboratory of Maternity and Neonatology Center of Monastir, Monastir, Tunisia

**Keywords:** Vector competence, Biting midge, *Leishmania* transmission

## Abstract

**Background:**

*Culicoides* (Diptera: Ceratopogonidae) species are known to be the vectors of Bluetongue virus and African Horses Sickness virus (AHSV) in different areas of the world. Nevertheless, other researchers have hypothesized that these arthropods could be involved in the transmission of other pathogens such as *Schmallenberg* virus, *Plasmodium* and *Leishmania* parasites. Identification of the *Culicoides*’ potential vector competence is crucial in understanding the worldwide *Culicoides*/*Leishmania* life cycle.

**Findings:**

Blood fed and parous females of biting midges *Culicoides* spp. were collected between 2009 and 2010 in Central Tunisia. DNA was extracted from individual blood fed *Culicoides* and used as a template in a genus-specific PCR. *Leishmania* DNA was detected in 14 *Culicoides imicola* specimens and one *Culicoides circumscriptus*. In a second step, parasite identification was performed based on a single copy Topo-isomerase II gene specific amplification and sequencing. *Leishmania infantum* was identified in two infected *Culicoides* spp.

**Conclusion:**

This is the first report of *Leishmania* DNA detection from naturally infected wild caught *Culicoides* spp. Our finding supports the assumption that *Culicoides* spp. are a potential vector for *L. infantum*.

## Findings

Biting midges of the genus *Culicoides* are known to be vectors of a wide range of pathogens, most notably arboviruses [1]. In early research on *Leishmania* transmission, *Culicoides* have been suspected to be vectors of *Leishmania donovani* in India [2]. However, since this date and until the beginning of the 20th century, no data were published concerning the potential involvement of biting midges in *Leishmania* transmission. Between 2004 and 2010, some researchers reported the natural infection of *Culicoides* biting midges by *Herpetomonas* and *Sergeia* kinetoplastid protozoa [3-5].

In 2011 Dougall *et al*. [6] reported the natural infection of *Forcypomyia* day-feeding midges with *Leishmania* using both molecular approach and microscopic detection of promastigotes in their gut. Recently, Seblova [7] have experimentally proved the susceptibility of reared *Culicoides nubeculosus* to infection with *Leishmania infantum*. All these previous studies have highlighted the potential role of midges in the *Leishmania* transmission.

Moreover, other studies have demonstrated the infection of domestic dogs (reservoirs of *L. infantum*) with BlueTongue Virus (BTV) in Morocco [8]. This implies that *Culicoides* can feed on canine hosts and thereby transmission of *L. infantum* by this arthropod genus is possible.

In our study, we emphasize the likelihood of *Leishmania* transmission via *Culicoides*.

Insects were collected between 2009 and 2010 in Central Tunisia, using two light traps types: home-made CDC (Center of Disease Control, Atlanta, USA) miniature and OVI (Onderstepoort Veterinary Institute) traps in rural areas. Traps were set before sunset and collected the next morning. All insects were collected in a beaker filled with 70% ethanol. Sampled insects were carried to the laboratory and *Culicoides* were separated from other insect genera. Females *Culicoides* specimen were divided into engorged (i.e. insects with a full or partial blood meal), parous and unfed midges. For each female specimen, wings and genitals were mounted for morphological identification using Chaker’s key [9].

Total DNA from each individual, blood fed or parous, biting midge abdomen and head, was extracted and PCR was performed to detect *Leishmania* DNA.

In the first step, DNA from all specimens (blood fed and parous) was tested using genus-specific PCR primers targeting a part of small subunit rRNA gene for the detection of *Leishmania* spp. infection according to the protocol of Spanakos *et al*. [10]. In the second step, DNA from positive specimens was re-analyzed by a second set of primers targeting a Topo-isomerase II single copy gene. PCR products from this last PCR were sequenced for identification of *Leishmania* to the species level according to the protocol of Haouas *et al*. [11].

In total, 259 biting midges of *Culicoides* (blood fed, n = 189; parous, n = 70) were tested for *Leishmania* spp. infection. Morphological identification showed that these tested midges belonged to the following species: *Culicoides imicola* (n = 196), *C. jumineri* (n = 35), *C. cataneii* (n = 3), *C. paolae* (n = 10), *C. newsteadi* (n = 10), *C. circumscriptus* (n = 3) and *C*. sp. (unidentified *Culicoides*) (n = 2). Among these collected samples, 15 *Culicoides* specimens were positive for *Leishmania* spp. DNA using genus-specific PCR primers. To confirm this result all positive PCR products were sequenced and sequences were blasted using Blastn algorithm against the “non-redundant” GenBank sequence database. These positive specimens belonged to *C. imicola* species (n = 14) and *C. circumscriptus* species (n = 1). All of them were engorged females and no parous female was positive for *Leishmania* spp. In the 15 positive specimens only two were positive for *Leishmania* Topo-isomerase II gene primer set. They correspond to *C. imicola* (n = 1) and *C. circumscriptus* (n = 1). The sequencing of these Topo-isomerase II positive PCR products confirmed the presence of *L. infantum* DNA in the abdomens of both *Culicoides* species. It is the first case in the world of *Leishmania* DNA detection from wild caught biting midges *Culicoides*. This preliminary finding highlights the potential role of this insect in *Leishmania* transmission. Our result corroborates the findings of Seblova *et al*. who have succeeded to experimentally infect reared *C. nubeculosus* with *L. infantum*[7].

It is noteworthy that even phlebotomine sand flies captured in the same area as biting midges were positive for *Leishmania* DNA in their mid gut. Among them three *Phlebotomus perniciosus* specimen were infected with *L. infantum*[12]. This result indicates that *L. infantum* could be transmitted by more than one arthropod genus. This assumption is supported by the studies of Coutinho *et al*. who have reported the presence of *L. infantum* DNA and promastigote forms in *Rhipicephalus sanguineus* (tick) and *Ctenocephalides felis felis* (fleas) respectively [13,14].

Despite infected biting midges are blood fed the hypothesis of contaminated blood meals could be excluded. Indeed, infected engorged females were also analyzed to identify their blood meal origin according to the protocol of Haouas *et al*. [11] and *Homo sapiens*, *Capra hircus* and *Gallus gallus* hosts were identified (unpublished data). These hosts are not known to be reservoirs of *Leishmania* in Tunisia. The presence of *L. infantum* DNA in *Culicoides* specimens fed on animals that are not regarded as reservoirs of the parasite may indicate that these female *Culicoides* would have taken their first blood meal from *Leishmania* reservoirs (the dog in the case of *L. infantum* in Tunisia). Then, these females would have taken a second blood meal (just before being captured) from uninfected hosts (human, goats or chicken).

Our preliminary findings raise important questions to solve in future epidemiological studies on the *Leishmania* life cycle. Therefore, to confirm the vector role of *Culicoides* in *Leishmania* transmission, studies should be accompanied by direct microscopic observations to confirm *Leishmania* development and survival.

## Conclusion

Our study reports for the first time the detection of *Leishmania* DNA in the abdomens of wild caught *Culicoides* spp. Nevertheless, further studies such as the isolation of the parasite and its iso-enzymatic identification are mandatory to confirm this preliminary result.

## Competing interests

The authors declare that they have no competing interests.

## Authors’ contributions

Conceived and designed the experiments: DS, HB, EC and NH. Performed the experiments: DS and LR. Drafted the manuscript: DS, NH, HB, and EC. Participated in field missions: DS, HB. All authors read and approved the final manuscript.
